# Addressing challenges along the ‘access cascade’ for new TB regimens

**DOI:** 10.5588/ijtldopen.25.0610

**Published:** 2026-03-13

**Authors:** J. Servello, P. de Colombani, L. Dall’Olio, G. Gargioni, M.C.B. Raviglione

**Affiliations:** Centre for Multidisciplinary Research in Health Science, Universita’ di Milano, Milan, Italy.

**Keywords:** tuberculosis, drug pipeline, UNITE4TB

## Abstract

This is a decisive moment with multiple new chemical entities progressing through the TB drug pipeline. Our review aims to contribute to policy discussion around these new TB treatments, primarily to increase the chance of successfully and rapidly adopting new regimens where they are most needed. Our analysis is based on: i) stakeholder engagement efforts undertaken in the context of UNITE4TB (a global clinical trial consortium for development of new TB drugs and regimens), ii) the outcomes of a special session of the UNITE4TB Annual Meeting 2024 in which representatives from several key stakeholder groups (pharmaceutical, clinical, research, regulatory, oversight, and advocacy) spoke on the topic of access to new TB regimens; and iii) a review of the literature. We propose a model for an ‘access cascade’ detailing the necessary steps from early research and development to the introduction of new regimens into clinical care. We then determined potential bottlenecks that might impede equitable access to new TB regimens globally, and conclude with recommended actions for stakeholders to take to overcome or mitigate the effects of these bottlenecks.

In 2023 an estimated 10.8 million people (10.1–11.7 million) fell ill with TB and 1.25 million (1.13–1.37 million) died due to TB worldwide. Of those who fell ill, 400,000 (360,000–440,000) had multidrug- or rifampicin-resistant (MDR/RR) forms of TB and 6.1% were people living with HIV (PLHIV).^1^ As the leading infectious killer, TB remains a major public health problem, especially in low- and middle-income countries (LMICs) where access to diagnostics and treatments is limited. Without appropriate treatment, approximately two thirds of individuals with infectious pulmonary TB die within 5 years of being diagnosed. With the treatments currently recommended by the WHO, about 90% of the people with drug-susceptible TB can be cured after a course of TB drugs lasting 4–6 months, while a smaller percentage of people with MDR and extensively drug-resistant (XDR) TB can be cured after an often longer, burdensome, and expensive course of treatment. Shorter, more effective therapies are expected to support the WHO *End TB strategy*^2^ and the achievement of its ambitious 2035 targets of reducing TB deaths and incidence rate, respectively, by 95% and 90%.

Although still insufficient to meet these targets, an unprecedented number of new chemical entities and treatment regimens for TB are advancing through the development pipeline.^3–5^ However, past experience shows that only 1 in 10 new chemical entities progresses from phase 1 to phase 3 clinical trials.^6^ High development costs and lengthy clinical trials make new drugs prohibitively expensive and unaffordable for many countries without international support, greatly limiting the uptake of the newer regimens recommended by the WHO.^7^ Even after their adoption into policy, the new treatments are often delayed due to the need for further supporting data and other logistical hurdles. As a result, TB therapies frequently remain unavailable for years, delaying timely access in the countries that need them most.^8^ Alongside the development of new TB treatments, we need to learn from past experiences and proactively address obstacles that may delay their rapid uptake. These obstacles are apparent from the slow and troubled uptake of drugs such as delamanid and bedaquiline,^9^ with the latter already facing emerging drug resistance worldwide.^10^ As witnessed during previous epidemics, especially HIV/AIDS, inequality in access to technology is one of the driving forces in the persisting high incidence of infectious diseases.^11,12^ Improving access is therefore not only a matter of equity but a necessary component of ending TB.

This review aims to contribute to the international dialogue on improving access to essential new TB treatments.^13^ Building on the stakeholder engagement being conducted by the Centre for Multidisciplinary Research in Health Science (MACH)^14^ of the Universita’ di Milano (UMIL), Italy, as part of the UNITE4TB Project,^15^ it attempts to provide an overview of challenges hindering access to new TB treatments and proposes actionable steps to accelerate their uptake in national health systems, including those that can be taken in preparation before the final results of large clinical trials are available.

## METHODS

This paper is based on a narrative review that integrated i) the outcomes of interviews and follow-up discussions with major stakeholders, ii) of a public round table session organised within the context of the UNITE4TB (Academia and Industry United Innovation and Treatment for Tuberculosis) annual meeting held in May 2024, and iii) a narrative literature review conducted to support a broader understanding of the topics raised. UNITE4TB is a public–private partnership with the goal of delivering novel phase 2 clinical trials that will accelerate the development of new TB drugs and regimens. The project focuses exclusively on treatments, which remained the focal point of the review.

### Stakeholder analysis

In 2022, our MACH team conducted a stakeholder analysis by mapping and interviewing 50 key stakeholders in the TB field (a selection of policymakers, national TB programmes [NTPs], financial supporters, and agencies at national and international levels) and participating in 4 key regional TB stakeholder events. Through analysis of the interviews and meeting outcomes, 25 distinct topics were identified, leading to the development of 10 key recommendations for the adoption of new treatment regimens. Selection of stakeholders and the ranking of these topics are detailed in the Supplementary Data to Villa et al.^16^ In 2023–2024, additional follow-up interviews were carried out with a select group of the above stakeholders. These two follow-up rounds of stakeholder engagement provided deeper insights into the main challenges in implementing each recommendation. Additionally, in May 2024, representatives from relevant organisations, involved in drug development, manufacture, licensing, procurement, as well as the development and implementation of health policy, were invited for further discussion on the topic of access at the UNITE4TB Annual Meeting.^17^ Those discussions prompted this narrative review.

### Narrative review

A narrative review of literature was conducted based on the topics highlighted throughout the stakeholder analysis. A two-step search on MEDLINE, PubMed, PMC (PubMed Central), and all journals indexed by Google Scholar was conducted first by using the keywords ‘research and development’, ‘patenting’, ‘manufacturing’, ‘registration’, ‘endorsement’, ‘inspection’, ‘reimbursement’, ‘pricing’, ‘procurement’, ‘funding’, ‘distribution’, ‘prescription’, ‘dispensing’, and ‘pharmacovigilance’, each in combination with ‘access’, ‘equity’, or ‘tuberculosis’. Additional searches were carried out for specific named strategies, challenges, and organisations. These searches were conducted from May 2024 to February 2025, with Supplementary Data added where relevant.

## THE ‘ACCESS CASCADE’ FOR TB MEDICINES

A novel ‘access cascade’ for new TB medicines was created by adapting the model proposed by Vogler et al.^18^ (Figure). It details the major steps from evidence generation through the negotiation of access and price to the uptake of new regimens in countries. Constraints in accessing new TB treatments may be more easily identified by following each step of this cascade. Access to new TB treatments which are suitable for patients and sustainable for health systems, while still incentivising early research and development, is the final result of policy decisions taken along a cascade of interventions that characterises the lifecycle of any pharmaceutical product. Beginning with basic research to identify new chemical entities and clinical trials to determine their safety and efficacy (often guided by WHO target profiles), new drugs and treatments are patented as premise for their marketing. The approval by stringent regulatory authorities opens the new drugs/treatments to manufacturing and pricing, which may be influenced by the endorsement of the new TB drugs/treatments by the WHO. The regulation of access to new drugs/treatments within countries depends on their registration with national/supranational authorities (which also may take WHO recommendation into account), the pricing, the revision of national policies, the procurement supply management (with domestic or external funding), and on delivery by the health providers who, adequately trained, are responsible for prescription, dispensing and patient support, pharmacovigilance, and operational research. The engagement of the people affected by TB, their communities, and civil society at large is key in many of these steps.

**Figure.**
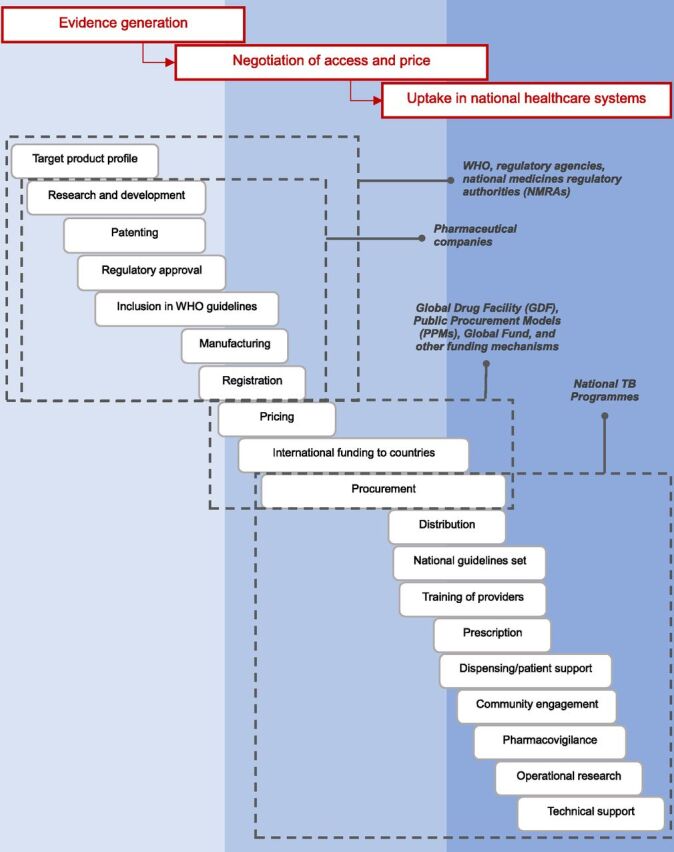
A model of the ‘access cascade’ for new TB treatment regimens.

## EVIDENCE GENERATION

### Strategic approaches for basic research and development

One of the six core functions of the WHO is ‘shaping the research agenda and stimulating the generation, translation and dissemination of valuable knowledge’.^19^ Consequently, the WHO has included ‘intensify research and innovation’ as one of the three pillars of its End TB Strategy^2^ and subsequently identified multiple target product profiles (TPPs),^20–22^ preferred product characteristics, and target regimen profiles to provide high-level information on the desired characteristics of the product, the target population, and the key endpoints for safety and efficacy evaluation. Furthermore, the WHO has issued a guidance on evidence generation that provides technical information on how to design pivotal clinical efficacy/validation trials with global access considerations in mind and assisting the production of suitable evidence for WHO future recommendations.^23^ The use of TPPs by pharmaceutical companies and medical device manufacturers has been steadily increasing since the first release of the International Council for Harmonization (ICH) guideline Q8 R2,^24^ and again following the launch of the Global Observatory on Health Research and Development^25^ in 2017. TPPs can help the development of new products around the end-user needs and be pursued together by international agencies, investors and donors, the pharmaceutical industry, and regulatory bodies.^26,27^ In TB, TPPs for biomarker assays may support the redefinition of patient outcomes and accelerate phase 2 and phase 3 clinical trials.^28^ The compliance with TPPs paves the way for new TB drugs/treatments to be recommended by the WHO, financed where needed by international funding mechanisms such as The Global Fund to Fight AIDS, Tuberculosis and Malaria (Global Fund), and registered in countries. Therefore, researchers are urged to respond to the challenge of ensuring that new TB drugs/treatments are not only fit for approval by a regulatory authority but also in ensuring their compliance with the WHO Guidance Development Group process and policies for country implementation.^29^

### Clinical trials

In a 2022 analysis comparing drug development for TB, HIV, and hepatitis C virus (HCV), a dramatic disparity in the number of phase 3 trials was observed, with fewer TB drugs in the pipeline. Moreover, the time from the first peer-reviewed publication reporting chemical activity against the pathogen to regulatory approval of the new drug ranged from 8 years (bedaquiline) to 19 years (pretomanid), while this stood at only 1.6 to 4.7 years for HIV and HCV drugs.^30^ This is within a context where research and development of drugs for ‘neglected diseases’ is already estimated to receive 10 times less investments than would be expected based on the attributable global disease burden.^31^

Despite significant progress in the discovery of novel compounds for TB treatment, challenges remain, particularly in securing sustainable funding for advanced clinical trials. Late-stage trials, especially during phase 3, often face uncertain funding, risking delays in the availability of new therapies. Collaborative initiatives and partnerships involving governments, research consortia, and global health institutions can play a vital role in addressing these gaps and ensuring steady progress. Incentivising manufacturers through public funding, therefore, can be a critical component to the initial stages of drug development. Public–private research consortia, including FAST-TB, ERA4TB, UNITE4TB, and the Tuberculosis Drug Accelerator have proved successful in engaging and partnering with pharmaceutical companies and developing both new TB chemical entities and regimens.^29,32^

A further issue to consider in early-stage research is the representativeness of the populations targeted for enrolment in trials. Several populations at higher risk of TB have not been engaged in major clinical trials, including people younger than 18 years, people aged 65 years or older, pregnant or lactating people, people with diabetes mellitus, and people with alcohol and/or other substance use disorders.^33^

### The European Medicines Agency and the US Food and Drug Administration (FDA)

The European Medicines Agency (EMA) has multiple regulatory tools for medicines which address unmet needs, including Conditional Marketing Authorization and Accelerated Assessment. The agency also encourages drug development through several initiatives including PRIME (‘priority medicines’) and the Innovation Task Force, which support schemes designed to promote early scientific dialogue with manufacturers.^34^ Within the European Union (EU), ‘orphan’ drugs are defined as medicines for the treatment of life-threatening or chronically debilitating conditions that affect no more than 5 people per 10,000 in a country’s population.^35^ Between 2015 and 2017, the annual share of orphan drugs among approvals by EMA ranged from 16% to 21%. It has been forecasted that by 2025 about 120 new orphan medicines will have been approved, resulting in a budgetary impact of approximately €22 billion.^18^ While TB drugs, including bedaquiline and delamanid, have benefitted from the EMA’s Accelerated Assessment which allowed NGOs to carry phase 3 trials concurrently with the review, phase 4 trials were required to expand their access to a wider population; therefore, the programme had only a limited effect.^36^ Similarly, the US Orphan Products Grant Program included four TB drugs as of 2023, and the *Mycobacterium tuberculosis* complex is included among the pathogens targeted by the Generating Antibiotic Incentives Now (GAIN) initiative, providing various benefits to manufacturers.^37^

A significant amount of time is required by the EMA to process new applications. In 2018, the EMA took an additional 60 days (median) to review new drugs in their standard procedure, and also took longer on average in their expedited programmes, in comparison to the FDA.^38^ The Clinical Trials Information System for registering clinical trials and submitting documentation to the EMA has been identified as a source of delay among researchers, who cite pricing and a lack of harmonisation as bottlenecks.^39^

As of 2024, the EMA is updating their guidance on adaptive clinical trial designs following consultation with statisticians and methodologists; their recommendations are of particular relevance for those research consortia employing adaptive trial designs in their development of TB drug regimens.^40^ Fast-track procedures are considered by both EMA and FDA for the development, evaluation, and registration of drugs to treat serious conditions and fill an unmet medical need, including the conditional approval on the basis of less comprehensive data than normally required, and likewise market authorisation to orphan drugs developed to treat rare medical conditions.^41^ In LMICs, registration may rely on the WHO Certificate for Pharmaceutical Products Moving in International Commerce and other product information provided by the Global Drug Facility (GDF) from a list of WHO pre-qualified manufacturers. Fast-track registration and waiver of registration fees should be applied on GDF drug donations.

### Inclusion in WHO guidelines

WHO may decide to provide formal recommendations for the consideration of new TB drugs/treatments in national policies and guidelines, which is especially important for LMICs.^42,43^ The inclusion of new treatments by the WHO in its evidence-based guidelines and the endorsement of their use is a fundamental condition for resource-poor countries willing to procure the innovation to receive financial support from international donors such as the Global Fund.^16^ Whether a medical innovation is included by the WHO in its guidelines depends on the Grading of Recommendations Assessment, Development and Evaluation (GRADE) approach by which the existing body of evidence is assessed.^44^ Through the GRADE approach, the WHO tries to ensure an assessment through an explicit and transparent process that minimises the risk of bias and considers the balance between potential benefits and harms of the new health interventions.

## NEGOTIATION OF ACCESS AND PRICE

### Patenting

Several strategies to overcoming challenges and potential bottlenecks in the patenting process have been put forward by researchers and piloted by organisations. For example, implementing a priority examination programme for patent applications can increase the number of patents granted and hasten timelines, as in the case of the introduction of the Federal Law 9279 in Brazil. Jurisprudence to allow use of patents without voluntary authorisation of the right holder, to allow research and development of a drug before patent expiration, and the introduction of laws to prevent evergreening have also been underlined as strategies. Flexibilities within existing trade agreements such as the Agreement on Trade-Related Aspects of Intellectual Property Rights (TRIPS) can allow exceptions for public health reasons.^45^ Once patents have been established, other strategies can be employed to ensure access. Patent holders have multiple routes to approaching technology transfer, allowing manufacturers to legally produce the patented TB drugs, such as voluntary licensing agreements, technology transfer contracts, and know-how sharing agreements. The importance of such agreements is evidenced by the Technology Transfer Agreement between Shionogi & Co Ltd. and the Global Antibiotic Research and Development Partnership (GARDP), which secured production of cefiderocol in 135 countries.^46^

Another strategy is to pool patents together. The Medicines Patents Pool (MPP) is a private initiative to promote access of anti-TB drugs in LMICs. The MedsPaL database provides information of intellectual property of drugs. Although the MPP was initially designed only for HIV drugs, since 2015 it has begun to include medicines for HCV and TB, functioning to negotiate with patent holders for drug licenses that allow generic companies to develop subsequent treatments, including fixed-dose combinations (FDCs) and paediatric formulations.^47^ It has been suggested through an income differential analysis that, while special policies and funding by international donors help to mitigate the effects of variations in patent regimes, entry of generics into the market can make a considerable difference in pricing of TB medicines (up to 69%).^48^

### Manufacturing

Manufacturing can have a profound impact on the availability of TB medicines, mainly through supply chain performance and the quality of the medicines produced. In a scoping review of the issues faced in supply chains of the WHO African region, manufacturing shortages were identified as a leading cause of poor drug availability, especially when encountered in combination with unanticipated spikes in demand and a lack of incentives to maintain stocks.^49^ An increased investment in local manufacturing, particularly in light of United States Agency for International Development (USAID) funding cuts, would help to mitigate these issues,^50^ as well as lower transportation costs, reduce dependency on international supply chains, and allow for quality control more tailored to regional needs. Recent investments of nearly US$50 million by Unitaid demonstrate this importance.^51^

Regarding the quality of medicines, a 2023 study of quality assurance and quality control (QA/QC) in 22 countries found that 15.2% of TB medications failed at least one quality test – though they caution that due to a paucity of data, the results are not necessarily generalisable globally.^52^ There are multiple ways to address such issues. Discovery research prioritising the stability and biological potency in real-world conditions could help to mitigate this risk. Technology transfer agreements can help to ensure acceptable quality standards by sharing detailed manufacturing protocols and quality control methods, and training by the original patent holder to the receiving manufacturer. Finally, the GDF also has the potential to expand its role here, having shown robust results in their QA/QC methods in all batches of TB medications procured from 2013 to 2017.^53^

### Pricing

Pricing is determined by a multitude of causes, such as patenting, costs in manufacturing, distribution, regulation, as well as provider and patient awareness. The influence of market demand is another major factor, and market shaping is the act of intervening on demand and supply imbalances along the product lifecycle.^54^ Such interventions can help to overcome pricing obstacles and ensure availability of products. Several approaches, including public–private partnerships for product development, increasing the number of manufacturers to guarantee volumes, pooled procurement, advance market commitments, regulatory incentives, and up-front payment, have been variously used in the past, such as in the cases of pooled procurement through the GDF^55^ and up-front payment from Unitaid, the Bill and Melinda Gates Foundation, USAID, and the Plan for AIDS Relief (PEPFAR) for GeneXpert diagnostics.^56,57^ A number of key enablers are recognised for an effective market shaping; among them, the pharmaceutical manufacturers creating access plans for LMICs before the market entry of the new chemical entity/treatment.^58^ While pharmaceutical companies increasingly develop access plans for new compounds, many of these plans lack comprehensive provisions for countries with the highest TB burden. Expanding the scope and inclusivity of access plans is essential to avoid exacerbating global health inequalities. In 2022, only 6 of the 20 major pharmaceutical companies surveyed had access plans in place for all its late-stage drug candidates and only 15% of them were considering LMICs.^59^ Ideally, access plans should also consider accessibility (registration with national regulatory authorities, awareness), affordability (tiered pricing based on the World Bank income classification),^60^ and availability (in-house manufacturing models, voluntary licenses).

### International funding mechanisms

Funding challenges for TB treatment are well documented.^61,62^ In many high-burden countries, TB prevention and control efforts depend heavily on external sources.^63^ In a study of 30 countries with a high burden of TB, researchers have found that just over half were primarily funded by government, with LMICs still largely dependent on financial aid.^64^ While funding for first-line drugs is largely derived from domestic sources within countries (around 80%), this need for external funding is still particularly acute for MDR-TB treatment. More recent global reports indicate a decline of US$ 1.2 billion in available domestic funding, and a slight increase of US$ 0.1 billion from international donors – mostly due to reductions in domestic funding in Brazil, the Russian Federation, India, China, and South Africa (BRICS) from 2019 to 2023.^1^ As of 2020, the Global Fund had disbursed US$ 10.3 billion for TB programmes around the world. These funds have considerably impacted the burden on national health programmes by assisting the treatment of more than 12 million TB patients, including those with MDR-TB.^65,66^

Recent cuts to donor funding threatens progress in TB control, with some models estimating hundreds of thousands of additional TB deaths between 2025 and 2035,^67^ creating an urgent need for an increase in domestic spending – especially in the form of results-based funding, health insurance schemes, and private sector collaborations.^68^ Other models include ‘blended financing’, debt-for-health swaps, catalytic and matching funds, and increased integration of TB services into primary health care.^69,70^ The current financing model in Indonesia has been cited as one example of success. Developed by the World Bank and the Badan Penyelenggara Jaminan Sosial Kesehatan (BPJS-K), it emphasises strategic purchasing and performance-based payments.^71,72^

When international assistance is provided, it is often in the form of lump-sum grants. Though these are disbursed over several years, this method of lump-sum payment can cause logistical issues, leading to widespread stockouts of life-saving drugs. There is a noted relationship between cumulative stockouts and receipt of Global Fund assistance from 2002 to 2013 in East Africa due to the unpredictable timing of the fund disbursement. These authors recommend that shifting some fund disbursements up-front may help to buffer stocks against future uncertainty.^73^ Such challenges have become more pronounced following the termination of foreign assistance through the USAID, as well as the reduction in funding of foreign assistance programmes at The President’s Emergency Plan for AIDS Relief (PEPFAR) and the US Centers for Disease Control and Prevention (CDC). Those organisations affected in South Africa report that a median 44% of activities have been suspended, including TB prevention and treatment operations.^74^ It has also been shown that such financial aid can be allocated unequally among different aspects of TB prevention and control; for example, public–private mix contributions may neglect critical functions of TB programmes such as notification and surveillance, especially in countries where TB has only been made a legally notifiable disease within the last few years.^75^

## UPTAKE IN NATIONAL HEALTH CARE SYSTEMS

### Registration by national medicines regulatory authorities

The registration with national medicines regulatory authorities (NMRAs) of new TB chemical entities and treatments is required in countries to ensure their safety, efficacy, and quality before entering into the market. Such evaluation and registration are pooled for the EU countries under EMA and for the USA by the FDA. Internationally, stringent regulatory authorities, or WHO-listed authorities, are those which have met internationally benchmarked standards for review, inspection, and clinical trial oversight via tools such as the WHO’s Global Benchmarking Tool. When a drug or treatment is approved by a stringent regulatory authority or WHO-listed authority, other countries may accept or fast-track approval, reducing the need for repeated dossier submissions and local trials. In contrast, where regulatory authorities lack recognition or capacity, companies must submit full dossiers separately and pay additional fees.^76^

The high cost of registration may discourage manufacturers to submit dossiers of product information in countries of low market interest because of their small population or low TB prevalence. Countries may grant a special permit to import and use unregistered drugs under humanitarian emergencies, rare or life-threatening diseases with no feasible licensed treatment (for individual patients), and clinical trials. Some countries grant registration to applicant manufactures only if additional evidence is produced from national clinical trials; for example, Japan and China request the conduction of clinical trials in the country of data from international clinical trials where their nationals have participated. India has eased, but not removed, similar regulatory requirements in the recent years.^77^

While the International Council for Harmonisation of Technical Requirements for Pharmaceuticals for Human Use (ICH) has helped to unify requirements in Europe, USA, and Japan, other regions of the world have no, new, or converting guidelines for drug registration. Harmonisation of requirements globally may help to reduce this duplication of labour and ease the burden in registering new products.^78^ Despite the principle in research ethics that burdens and benefits of research should be equitably distributed among the people affected by it,^79^ LMICs hosting clinical trials have been shown to benefit least in terms of market access. This can be due to hurdles presented in approval from NMRAs due to marketing of drugs to patient groups not well represented in the pre-approval trials (such as children, women, the elderly, or minority groups), drug manufacturers failing to apply for local marketing approval, as well as the regulatory complexity and slower review times in LMICs.^80^ WHO prequalification serves as an important system of independent evaluation and approval of medicines, allows for the setting of international benchmarks, and complements the NMRAs. This is particularly true in LMICs, where prequalification allows for the fast-tracking of certain products and prequalification assessments can help to guide local registration.

A frequently cited cause for the delay in the adoption of newer drugs such as bedaquiline and delamanid is the lack of national regulatory approval.^81^ Manufacturers may be unwilling to file for regulatory approval for several reasons, for example, due to the calculated costs of approval versus the small available market. The lack of availability of rifapentine in Europe, despite its Orphan Designation, illustrates this issue.^82^ Many NMRAs also have requirements that the drug is tested locally to ensure suitability among their own patients.^83^ While most countries with a high burden of TB have mechanisms in place to allow for access to medicines prior to or during regulatory approval, for example, through import waivers, this only benefits a small number of patients. Previous reports have shown that non-registration of new TB drugs has historically been one of the major barriers for their uptake, with the Stop TB Partnership showing in 2015 that only 28% of the surveyed countries with a high burden of multidrug-resistant TB (MDR-TB) had registered bedaquiline and only 12% had registered delamanid.^84^ Additionally, while previous TB drugs have been approved conditionally after phase 2B trials due to the urgency and lack of effective regimens against DR-TB, a lack of phase 3 trial data can prove to be a major hurdle.^81^ Even in countries of relatively low TB burden, however, the registration of TB drugs (or lack thereof) can have a decisive effect on the availability and quality of treatments. Médecins Sans Frontières highlight such a case in their commentary on the lack of all-oral and newer regimens for drug-resistant TB in Poland and Slovakia, driven in part by the needs of Ukrainian refugees.^85^ A huge cost differential for MDR-TB treatments exists across Europe due to patenting, the comparatively long length of treatment, and a lack of access to discounted pricing mechanisms.^86^

Another possible route to ensure access is ‘compassionate use’, typically referring to programmes in which a physician requests a drug for a specific individual patient, usually appealing directly to the manufacturer.^87^ While most health authorities in countries with a high burden of MDR-TB are aware of this mechanism,^84^ this approach has a number of clear limitations, for example, it can only be offered to a limited number of patients, there may be a fee to the regulatory agency to process the application in certain countries, and the process for the importation for unregistered drugs can be complex.^9^

### National policy and guidelines

National guidelines provide the bases for all training that follows a change in TB treatment policy, and therefore must be changed as early as it is feasible in the ‘access cascade’. Covering all administrative units may take several months or years depending on the size of the country.^88^ Policy changes can outpace the implementation of a previous cycle of recommendations, posing a serious problem when successive recommendations are made. A lack of incorporation of WHO guidelines into national health policy has been shown to diminish the quality of TB treatment and preparedness for TB-related emergencies, as observed in the response to the needs of refugees arriving in Poland and Slovakia from Ukraine in 2022.^85^

National policymakers may also be reluctant to adopt new regimens based on concerns about adverse events and other potential complications associated with new drugs.^89,90^ Even when a new drug has been approved by the NMRA, policymakers may still wish to see additional data from local populations ensuring the safety and efficacy of a new regimen.^83^ In order to meet this precaution, engagement of NTPs and policy recommendation institutions from an early stage allows for their active input on trial elements, for example, in the definition of outcomes in the clinical trials, the identification of appropriate efficacy, safety, and tolerability measures, as well as the opportunity to extend certain phases of the trial so that follow-up matches the length of intended total duration of the new treatment.^91^

### Procurement

Public procurement models (PPMs) may take the form of national-centralised PPMs, regional-centralised PPMs, facility-based PPMs, or cross-country collaborative PPMs. While most countries use more than one form of PPM, depending on the type of medicines and sector, the majority of procurement happens at the facility level, for example, an individual hospital procuring medicines through a local trader. For those drugs of public health relevance, such as HIV/AIDS, vaccines, and sometimes anti-TB drugs, this usually happens at the national-centralised level.^92^ As discussed in the section on Evidence generation, pharmaceutical companies may have managed entry agreements or access plans in place when developing TB drugs. When a country is not covered by such pricing agreements, TB drugs may be procured from the GDF, particularly in LMICs. In order to best facilitate procurement, GDF offers a pre-payment mechanism for domestic procurement, registration support, fee waivers (where applicable), review and production planning, and access to their Strategic Rotating Stockpile.^93^

Mechanisms such as pooled procurement through initiatives like those within the GDF have proven effective in negotiating competitive prices and ensuring steady supply chains. Exploring innovative approaches, such as regionally tailored procurement models or bilateral agreements, could help bridge this gap. Even with such services, procurement can present a problem for the uptake of new TB drugs,^94,95^ especially in those countries with limited manufacturing capacity.^96,97^ Stockouts are a recognised challenge in initiating and continuing TB therapy, from preventive therapy to second-line treatments.^98,99^ Processes for procurement are increasingly integrated within public health systems globally, including for TB drugs, meaning that discussions must be held at a national level to agree on new guidelines before the adoption of a new TB regimen.

The GDF commands a considerable part of the market, providing first-line drugs for an estimated 35% of TB cases occurring worldwide.^100^ Historically, they have played a crucial role in negotiating drug prices and have done so for MDR-TB regimens since 2010,^101^ as well as creating an accelerated pathways for access to medicines such as the Bedaquiline Donation Program which procured more than 33,000 courses across 80 countries as of 2020.^102^ However, several countries have limitations in procuring TB drugs through GDF. This has previously been the case for China, Russia, and South Africa with regard to delamanid,^81^ which together make up 26% of the global drug-resistant TB burden.^103^ Other countries have limitations in procuring TB drugs through GDF because of its procedures for direct procurement which exclude international bidding and require advance payment, which both present challenges for certain countries, particularly those within the EU.^104–106^

FDCs have recognised advantages and disadvantages, as found through both stakeholder engagement and in editorial literature. While it is recognised that FDCs potentially reduce adverse drug reactions (ADRs) by minimising the risk of selective drug intake and increase rates of adherence, there is also a possible increase chance of drug interactions, reduced clinical effectiveness in some cases, and higher pricing due to an increased need for quality control and the additional regulatory requirements for combined products. Some of these concerns could be alleviated with physician and patient education, the development of quality indicators around their use, and ensuring realistic pricing with pharmaceutical companies during the complex licensing process.^107^ It is also worth noting that FDCs have generally become more affordable over time, also through the introduction of generic formulations.^108^ Multiple examples exist of pro-access licensing agreements with wide geographic scope for FDCs, especially with regard to HIV/AIDS treatments, that could serve as a model for future TB regimens should they be combined into an FDC. For example, GSK (through ViiV Healthcare) licenses abacavir and dolutegravir via the MPP, making generic production available across most low- and middle-income countries; similarly, Merck & Co. has a broader commitment not to file for patents in low-income countries.^109^

### Distribution

Distribution is the last part of the drug management process^110^ and carries a number of potential bottlenecks. National governments have a critical role in addressing cost barriers of distribution. Reducing import tariffs, streamlining regulatory approval processes, and engaging in public–private partnerships can significantly lower costs and improve the availability of new TB drugs. By addressing these systemic issues, stakeholders can work toward a pricing model that ensures sustainable and equitable access to new TB treatments across all settings. According to the World Trade Organization, the highest average import tariffs are found in South Asia and Latin America – India, Nepal, and Pakistan are among the most expensive and rank among the countries with the most severe TB burden in the world. In a global survey of tariffs, researchers have urged governments to take low-cost steps that could improve access to health care, including reducing tariffs and reducing time for patent examination.^111^

In addition to the role that funders can play by weighting the disbursement ‘up-front’, allowing for the purchase of a buffer inventory, national pharmaceutical supply agencies should take care to avoid delays in delivery which result in stockouts.^99^ Therefore, when offering financial assistance for procurement, the Global Fund can play a critical role by disbursing more of the funding up-front, thereby allowing countries to create a buffer stock of drugs and mitigate the risk of future stockouts or supply disruptions. At a district and local level, poor storage practices are known to have an effect on the efficacy of TB drugs,^112^ and therefore it is essential to maintain adherence to Good Storage Practices.^113^ The WHO recommends that, for optimal storage conditions, temperatures should be maintained between 15°C and 25°C with a relative humidity below 60% to ensure efficacy.^114^ Electronic systems like QuanTB and e-TB Manager also play a role in enhancing inventory management by tracking drug consumption, monitoring expiry dates, and providing alerts for potential stockouts or imminent expirations.

### Training and re-training of clinical staff

A change in the recommended guidelines for TB treatment can result in an upheaval of routine for national TB programmes; staff re-training, as well as the adjustment of administrative tools and medical records, must be deployed in order to adapt to a new treatment duration and strategy. Studies of health system factors in the management of MDR-TB in EU countries have illustrated that, while short courses are organised for physicians and nurses, one of the most important sources of training is periodic review of the medical records and weekly clinical meetings among staff to discuss the patients.^115^ In a 2019 pilot study in Zimbabwe, researchers found that district staff analysing their own local TB data and holding ‘data-driven’ supervision and performance review meetings led to an improvement in the quality of patient care, allowing them to set priorities and take better ‘ownership’ of TB services.^116^ A virtual technical support programme undertaken in 2021 which used a point-of-care continuing education model with ancillary live lectures and case-based conferences was shown to be an effective means of reaching a large number of clinicians globally.^117^

### Dispensing treatment and patient support

A major component of access to treatment is ensuring that patients can navigate the existing health systems within their own countries to obtain the correct diagnosis and be set on the right care pathway. There is substantial evidence offered in TB literature that barriers to treatment still exist, including distances to clinics, costs associated with travelling and undergoing lengthy treatment regimens, and the need to visit health centres daily.^118^ Financial hardship also appears repeatedly in studies globally.^119^ Financial support throughout TB treatment is recognised overwhelmingly as a positive by patients, leading to faster diagnosis and more favourable adherence patterns.^66^ Other forms of material support, such as nutritional supplementation, have also been shown to be highly effective means of reducing TB incidence and improving treatment outcomes.^120^

In several countries with a high incidence of TB, patients are drawn to better client experiences and therefore are more likely to visit private clinics, despite being more expensive and frequent deviation from national TB guidelines.^121^ ‘Patient navigators’ (i.e., guides who assist the most vulnerable patients through any potential barriers to health care, such as necessary identification, understanding the system of referrals, and reaching appointments) have been identified as a highly effective intervention in terms of helping patients to initiate treatment.^122^

Monitoring and evaluation (M&E) systems are pivotal to ensuring the successful implementation of new TB regimens within national health programmes. Well-designed M&E frameworks enable the timely identification and resolution of key challenges, such as stockouts, suboptimal drug use, and gaps in health care worker training. These systems provide essential feedback on the effectiveness of new treatments and help guide corrective actions.

There are a multitude of ways to support patient adherence. The use of visual communication tools in Indonesia, as administered by caregivers, was shown to increase MDR-TB treatment success rates and was adopted as part of the national strategy.^123^ Digital adherence technologies, such as video-based directly observed treatment and electronic pillboxes, have shown promise in improving patient adherence to TB treatment regimens, though differentiated care among certain groups such as PLHIV and separated/single-living patients may be necessary.^124^ Other determinants of low adherence must be taken into consideration, as well, such as educational level, poor housing, certain occupations, and alcohol abuse.^125^ In high-income settings, psychosocial factors are understudied and may play an outsized role in patient adherence.^126^ Special considerations should be made depending on the demography of the patient, as in the case of children and young adults, who benefit considerably from peer-support networks and the use of digital technologies.^127^ Electronic dispensers of drugs can allow patients to receive pre-packaged doses while sending real-time adherence data to central systems, as shown in South Africa.^128^

Primary health care also has a role to play in improving access at the patient-support level. Strong evidence, including comprehensive meta-analyses, have shown that outreach clinics can increase TB case detection where there is a high prevalence of undiagnosed TB, comparing favourably to house-to-house screening in some settings.^129^

### Community and civil society engagement

In addition to the financial and technical support offered by international organisations, people affected by TB, communities, and civil society organisations can play important roles in adopting new TB treatments, enhancing access to diagnosis and treatment, supporting treatment adherence, and strengthening pharmacovigilance by advocating for adequate reporting systems and representing patients in committees evaluating the safety of new drug policies. Effective implementation of new TB treatment policies require not only the endorsement of community organisations at national level but also building community capacity at the grassroot, providing significant support to both health staff and patients,^88^ as well as providing an invaluable means of decentralising care in communities suffering from higher burdens of MDR-TB.^130^ Civil Society Organisations have been recognised as a critical category of actors in the introduction of new TB drugs going forward by international agencies such as Unitaid, who emphasise their role in renewed efforts in addressing MDR-TB.^131^

Public information and education have the potential to profoundly shape prevalence of TB and adherence to treatment. Mass media strategies have also been shown to have a considerable effect in India according to mathematical models simulating awareness campaigns resulting in human behavioural changes to avoid disease transmission risk.^132^ Awareness of new TB drugs can help to instigate policy change via activism and the generation of demand.

### Pharmacovigilance

With the introduction of BPaL(M), the majority of TB patients are on treatment for up to 6 months in both drug-susceptible and MDR-TB. However, treatment can last longer where these regimens are inappropriate or unavailable. The length of TB regimens may also be associated with ADRs, many of which can be severe in nature. Aside from posing a danger to patient in and of themselves, ADRs can also interrupt vital TB treatment. Furthermore, pharmacovigilance helps to inform rational and effective clinical decisions of health care workers during treatment. This becomes especially important in complex MDR-TB regimens and where TB treatment is given concomitantly with antiretroviral therapy. The WHO handbook on pharmacovigilance in TB treatment identifies three main methods of pharmacovigilance: spontaneous reporting, targeted spontaneous reporting, which are appropriate for routine monitoring in NTPs, and cohort event monitoring, which is an active form of surveillance undertaken in a similar manner to an epidemiological cohort study.^133^ These methods can be supported with monitoring systems such as the Active Drug-Safety Monitoring and Management system, developed by the WHO, which is used for tracking and managing side effects for new or repurposed TB drugs, especially in patients with MDR- and XDR-TB. Such systems help to maintain adherence throughout complex treatment regimens and generate systematic safety data.^134^ In 2021, an evaluation of TB pharmacovigilance activities in sub-Saharan Africa identified a lack of clarity about roles and responsibilities leading to underreporting of ADRs, concluding that this can be avoided for new drugs introduced through public health programmes via legal guidance and information sharing with the NMRA.^135^

### Operational and implementation research

The importance of the role of operational and implementation research in evaluating novel regimens, particularly the incorporation of new drugs into more tolerable and practicable regimens, has been well-established.^136^ Operational research also allows the assessment of those parameters which are crucial in setting new national guidelines, including the acceptability and feasibility of treatment, logistics, and cost-effectiveness of the new regimen. It has also been shown that when recommendations are made for new treatments based on low certainty of evidence, additional research becomes essential to test the merits of new standards of care and can generate the necessary data post-licensure to continuously update policies, as in the case of recent WHO guidelines to treat MDR-TB.^91^

### International technical support

Attributed in combination to the COVID-19 pandemic, the effects of persistent ‘brain drain’ in many regions, and a lack of central funds to TB programmes,^137,138^ shortages in health care workforce are an increasingly recognised problem across the world, with several countries of high TB burden reaching a crisis stage. When necessary, international technical support can help to alleviate this shortfall and strengthen the health systems necessary for delivering TB treatment. While ‘bundling’ various aspects of technical support together has been a challenge of technical agencies in the past, it is seen as a considerable benefit to national policy makers. In order to measure the effectiveness of technical support programmes, researchers have recommended adopting standard definitions of technical support activities and stating specific aims at the outset of programmes.^139^

## DISCUSSION

This review utilises the 10 key recommendations formulated in the context of stakeholder discussions related to the experiences of a large clinical trial network such as UNITE4TB to identify the main challenges for future implementation of regimen innovations emerging from this and similar trials. While these challenges are complex, multiple actors within the ‘access cascade’ can help alleviate them and ensure the rational and rapid uptake of new drugs as they emerge from the clinical trial pipeline. The goal of this review is to offer a model of the TB drug ‘access cascade’ along with substantive and tangible actions to specific stakeholders so that new TB drug regimens can be rationally adopted. The conclusions offer a range of actions for the various stakeholders involved to ensure this end. While these are ordered under the headings of the 10 key recommendations, the timing of this paper lends itself especially to highlighting the actions listed under recommendation 2, that is, those actions regarding determining affordability of new regimens, should they show efficacy and safety in trials. All stakeholder groups are active in dialogues among the research consortia, and we hope that our description of the ‘access cascade’ allows the facilitation of concrete actions by each.

Limitations of this review include an exclusive focus on English language publications, and, given the breadth of the subject, its non-systematic approach and variety of literature cited leaves openings for more comprehensive research on each topic. This broad scope, however, also allowed for the creation of a model that could facilitate policy dialogue and serve as a framework to identify further specific actions that can be undertaken across stakeholders. Further strengths include the extensive feedback of stakeholders as a primary source, which underpinned further exploration in the literature. Regarding the stakeholder interviews which also informed this work, one additional consideration is the selection process for follow-up interviews, which prioritised stakeholders by levels of policy influence – though perspectives external to the UNITE4TB project were still captured through the inclusion of global stakeholders, especially NTPs.^16^

## IMPLEMENTING PROPOSED CHANGES

For any of the proposed changes to be successfully implemented, each stakeholder group must take the following into consideration (see Table):

- A situation analysis: a thorough assessment of the current landscape to identify gaps and opportunities for improvement in the ‘access cascade’.
- A presentation and discussion of proposed changes: clear communication of the proposed changes and an inclusive discussion on how to implement them, ensuring that all stakeholders are aligned on goals and processes.
- Impact assessment: an evaluation of the potential impact of the changes on current operations and the broader health system, identifying any challenges or obstacles that need to be addressed.
- Contextualised recommendations: tailoring recommendations to the specific context of each country or region, acknowledging that different settings may require different approaches to effectively overcome the identified barriers.

**Table.** The 10 key recommendations^16^ and specific steps along the access cascade to overcome potential obstacles.

Key recommendations	Specific actions in the ‘access cascade’
1. Ensure that clinical trial outcomes meet WHO’s requirements to develop policies and guidelines.	•Incorporating the Target Regimen Profiles (TRPs): Product developers and governments should tailor trial specifications to the TRPs provided by the WHO to ensure usability and access to funding and procurement agreements.
2. Pursue strategic discussions to make the costs of new treatment regimens, drug susceptibility test, and other tests affordable to low- and middle-income countries to ensure equitable access.	•Development of access plans: Pharmaceutical companies should prioritise the creation of access plans for phase 2 and 3 trials to ensure that access strategies are in place before a drug is approved.
	•Government action on tariffs: National governments should reduce or eliminate import tariffs on TB medicines to lower the cost of new regimens and improve affordability in high-burden countries.
	•Ensuring universal availability of drugs in regional tenders by the Global Drug Facility (GDF): The GDF should expand its regional tendering programmes for all new drugs to include high MDR-TB burden, ensuring that drugs are accessible to the most affected populations.
	•Up-front funding from Global Fund: The Global Fund should consider disbursing more funds up-front to allow countries to build buffer stocks and prevent drug shortages.
3. Consider the development of fixed-dose combinations (FDCs) for regulatory submission as soon as a new regimen is approved.	•Ensure rational clinical use of FDCs: National health authorities should introduce proper physician and patient education specifically for FDCs where they are introduced, and to develop quality indicators around their use.
	•Tailored access plans for FDCs: While there is no universal approach for FDCs, pharmaceuticals should consider licensing drugs through the Medicines Patents Pool (MPP) and/or pro-access terms in their licensing agreements.
4. Strengthen coordination among TB clinical trial research consortia to minimise duplication of research and development efforts.	•Adherence to Guidance on Evidence Generation (GEG): Product developers and governments should follow the GEG provided by the WHO to ensure trial efficiency, as well as future access to funding and procurement agreements.
5. Engage major international stakeholders to facilitate dialogue among research consortia, coordinate efforts, and address bottlenecks in TB drug research, development, and policy implementation.	•Early engagement with National Medicines Regulatory Authorities (NMRAs): Clinical trial consortia and investigators should initiate early discussion with NMRAs to ensure that the regulatory pathway is clear and aligned with global access goals.
	•Urgent submission of dossiers to NMRAs: For drugs with urgent public health need, pharmaceutical companies should fast-track the submission of regulatory dossiers to NMRAs to expedite approval and availability.
6. Carefully select clinical trial sites that include vulnerable and high-risk populations.	•Collaboration with civil society: Pharmaceutical companies and international organisations should work closely with civil society to ensure that the voices of affected communities are considered in the planning and implementation of TB treatment programmes.
7. Enhance engagement of national TB programmes (NTPs) in the conduct of clinical trials to ensure proper site selection and build local research capacity.	•Consultation and input in evidence generation: Product developers should consult NTPs during the earliest possible stage of the trial, since they can play an invaluable role in defining trial parameters in a way that ensures the new regimens acceptability and usefulness.
	•Employ innovative educational methods in (re-)training staff: National TB programmes should encourage district staff to ‘take ownership’ of local TB data where appropriate and deploy resources to develop a continuing education model with virtual sessions and/or ancillary live lectures.
8. Support national TB programmes in identifying bottlenecks for the rapid uptake of new regimens.	•Maintain research activities post-licensure: Research consortia should continue activities following the introduction of new regimens beyond pharmacovigilance and actively participate in operational research.
	•Delineate clear institutional roles in pharmacovigilance: Public health authorities must collaborate with NMRAs to establish effective reporting systems for adverse drug reactions to ensure the safety and efficacy of new regimens as they are introduced on a wider scale.
9. Advocate with investors and donors for adequate and stable funding for advanced clinical trial phases.	•Commitment of national and international funding bodies: Incentivising product developers and pharmaceuticals through public–private partnerships has been shown to be an effective means of developing new chemical entities; a similar approach from these funders would help to ensure trials continue to phase 3 and beyond.
10. Encourage effective, regular, and timely information sharing with stakeholders on relevant scientific discoveries emerging from trials.	•Early engagement with national health authorities and NTPs: Research consortia are responsible for disseminating the most recent developments in their trials to stakeholders and ensure that their feedback is taken into account throughout clinical development.
	•Tailored communications plans in countries where clinical trials are held: Stakeholder feedback indicates that national health authorities and principal investigators would benefit from a tailored communications plan to encourage their collaboration and to actively involve NTPs where possible.

## CONCLUSION

Specific actions to take in the ‘access cascade’ were identified by this narrative review to overcome the bottlenecks detected throughout stakeholder engagement. For any of the proposed changes to be successfully implemented, each stakeholder group must take into consideration the above-mentioned recommendations.
